# Supplementary material for: Towards unified AI-driven fracture mechanics: the extended deep energy method (XDEM)
Source: Nat Commun. 2026 Aug 15;17:8492. doi: 10.1038/s41467-026-76748-1 (PMC13484471; doi:10.1038/s41467-026-76748-1)
Supplement: Supplementary file 3 [file 41467_2026_76748_MOESM3_ESM.zip › pic/Results/Stress_intensity_factor/Cross/XDEM/compare_sy_independent.pdf]

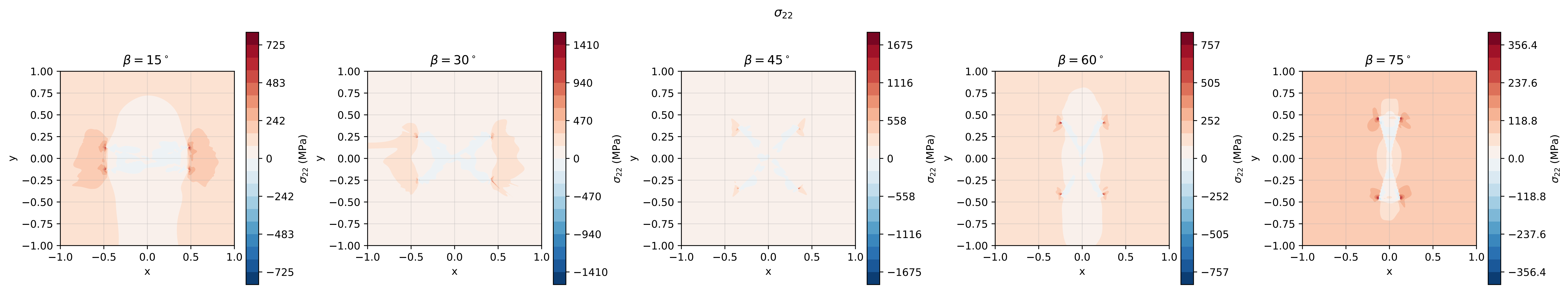

σ_{22}
$\beta = 15^\circ$
$\beta = 30^\circ$
$\beta = 45^\circ$
$\beta = 60^\circ$
$\beta = 75^\circ$
x
y
σ_{22} (MPa)
725
483
242
0
−242
−483
−725
1410
940
470
−470
−940
−1410
1675
1116
558
−558
−1116
−1675
757
505
252
−252
−505
−757
356.4
237.6
118.8
0.0
−118.8
−237.6
−356.4
